# A systems level approach to temporal expression dynamics in Drosophila reveals clusters of long term memory genes

**DOI:** 10.1371/journal.pgen.1007054

**Published:** 2017-10-30

**Authors:** Julianna Bozler, Balint Z. Kacsoh, Hao Chen, William E. Theurkauf, Zhiping Weng, Giovanni Bosco

**Affiliations:** 1 Department of Molecular and Systems Biology, Geisel School of Medicine at Dartmouth, Hanover, NH, United States of America; 2 Bioinformatics Program, Boston University, Boston, MA, United States of America; 3 Program in Bioinformatics and Integrative Biology, University of Massachusetts Medical School, Worcester, MA, United States of America; 4 Program in Molecular Medicine, University of Massachusetts Medical School, Worcester, MA, United States of America; Stanford University School of Medicine, UNITED STATES

## Abstract

The ability to integrate experiential information and recall it in the form of memory is observed in a wide range of taxa, and is a hallmark of highly derived nervous systems. Storage of past experiences is critical for adaptive behaviors that anticipate both adverse and positive environmental factors. The process of memory formation and consolidation involve many synchronized biological events including gene transcription, protein modification, and intracellular trafficking: However, many of these molecular mechanisms remain illusive. With Drosophila as a model system we use a nonassociative memory paradigm and a systems level approach to uncover novel transcriptional patterns. RNA sequencing of Drosophila heads during and after memory formation identified a number of novel memory genes. Tracking the dynamic expression of these genes over time revealed complex gene networks involved in long term memory. In particular, this study focuses on two functional gene clusters of signal peptides and proteases. Bioinformatics network analysis and prediction in combination with high-throughput RNA sequencing identified previously unknown memory genes, which when genetically knocked down resulted in behaviorally validated memory defects.

## Author summary

Long term memory formation is a complex process, and at different stages, requires regulation of transcription and protein synthesis. In a novel learning and memory paradigm, we examined transcriptional changes in the fly brain during and after memory formation. With RNA sequencing, we captured transcriptional waves of numerous genes previously not associated with learning and memory. Genes in the functional groups of proteases, signal peptides, and immunity, were selectively tested and behaviorally validated as memory genes. Placed into a large context and with computational methods, this work presents novel gene networks that may be linked to the learning and memory process.

## Introduction

The ability to form a memory is one of the hallmarks of the advanced nervous system. This capacity to learn from, and remember, past experiences is a critical attribute to many eukaryotes. It is this function that allows organisms to meet the various demands and challenges of a changing and stochastic world: The interruption of these processes is associated with devastating illnesses, such as Alzheimer’s Disease and Huntington’s Disease, amongst others. Given the importance of this facility, it is vital for us to understand the basic biological mechanisms behind the complex process of learning and memory.

A traditional view of learning and memory involves two phases, acquisition and retention. However, decades of investigation have revealed this to be an oversimplification, and instead, a variety of studies point to the existence of distinct features for learning, short term memory (STM), long term memory (LTM), memory consolidation, and memory retrieval [1–4]. Differences in mechanism and neural circuitry for these functions point to a degree of autonomy for these related processes [5,6]. For instance, it has been suggested that disruption in memory retrieval may be a pathology distinct from other memory impairments [7]. This notion is further supported by the identification of discrete waves of transcriptional activity and protein synthesis associating spatially and temporally with the various learning and memory processes [8,9].

One convention in the field is the distinction between short term and long term memory. Intrinsic to the process of STM is the transient nature of the memory. Mechanistically it relies predominantly on reversible processes, such as protein modifications that alter synaptic function [3]. Altered function of the synapse is not persistent in STM, lasting from minutes to several hours. Additionally, it is functionally unique in that STM can respond rapidly to stimulus and is generally characterized by being independent of *de novo* protein synthesis [10]. Alternatively, LTM relies on the time and energy consuming processes of transcription and translation. Formation of LTM creates persistent and stable alterations of the synapse including both changes in synaptic connections, as well as synaptic potentiation. These changes can last for hours to weeks and are a combined result of transcription factor activation, protein synthesis, and synaptic protein reorganization [11,12].

Dynamic gene transcription is central to synaptic plasticity, with distinct waves of gene expression defining the various phases of LTM formation [2,13,14]. Immediate early gene (IEG) transcription occurs rapidly in response to neuronal activity. Within minutes, key IEGs are up regulate. This rapid response to stimulus is made possible by chromatin accessibility and de novo protein synthesis independence [15,16]. In mammalian systems IEGs are enriched for transcription factors and are required for triggering later transcriptional waves [17,18]. In Drosophila, these activity-regulated genes are less well characterized; although studies indicate that the general dynamics of expression persist these ARGs appear to include a more functionally diverse set of genes [19]. Later waves of gene transcription encode key memory genes responsible for neural plasticity and memory establishment [20]. In general, the specific activity-related and subsequent target genes that change in expression seem to be paradigm specific [18,21]. In addition, these subsequent transcriptional waves are classified as either cycloheximide sensitive or cycloheximide insensitive, pointing to multiple mechanisms of transcriptional regulation that are distinguished by requiring new protein synthesis [22,23].

The identification of individual memory genes, such as Fragile-X mental retardation gene 1 (FMR1) and rutabaga (adenylate cyclase), has enhanced our knowledge of specific molecular processes in the neurons [24,25]. However, genes are likely acting in complex networks rather than having a singular effect on neuron function. In this regard, much remains unclear and a global picture of gene expression networks has not yet been established. In part, progress has been hindered by the technical challenges associated with traditional learning and memory paradigms. In particular, the high degree of variability between individuals and tissue types has created roadblocks to the systems level analysis common in other fields.

Classical studies involving associative learning paradigms and courtship rejection have identified critical learning and memory circuits in the Drosophila brain. The mushroom body (MB), considered the learning and memory center in Drosophila, consolidates the multiple sensory inputs critical to learning and memory formation [1,26–28]. This study utilizes an alternative approach and recently developed Drosophila LTM paradigm involving the endoparasitoid wasp, *Leptopilina heterotoma*. These wasps are a natural predator of Drosophila larvae. Although adult flies are not at risk of parasitism, female flies have an innate response to the presence of these wasps, resulting in a cascade of behavioral changes dependent on LTM formation [29,30]. Advantages to this paradigm include the robust response to the predator, and the persistence of this response across several days. We use this system to explore global changes in transcription over the course of memory formation. With a systems level approach of combining molecular, behavioral and computational methods allowed us to discover several previously unidentified LTM genes. In addition, by using a bioinformatics methodology we begin to place these genes in a larger context that distinguishes LTM formation from LTM maintenance.

## Results

### Memory formation

**Fig 1:**
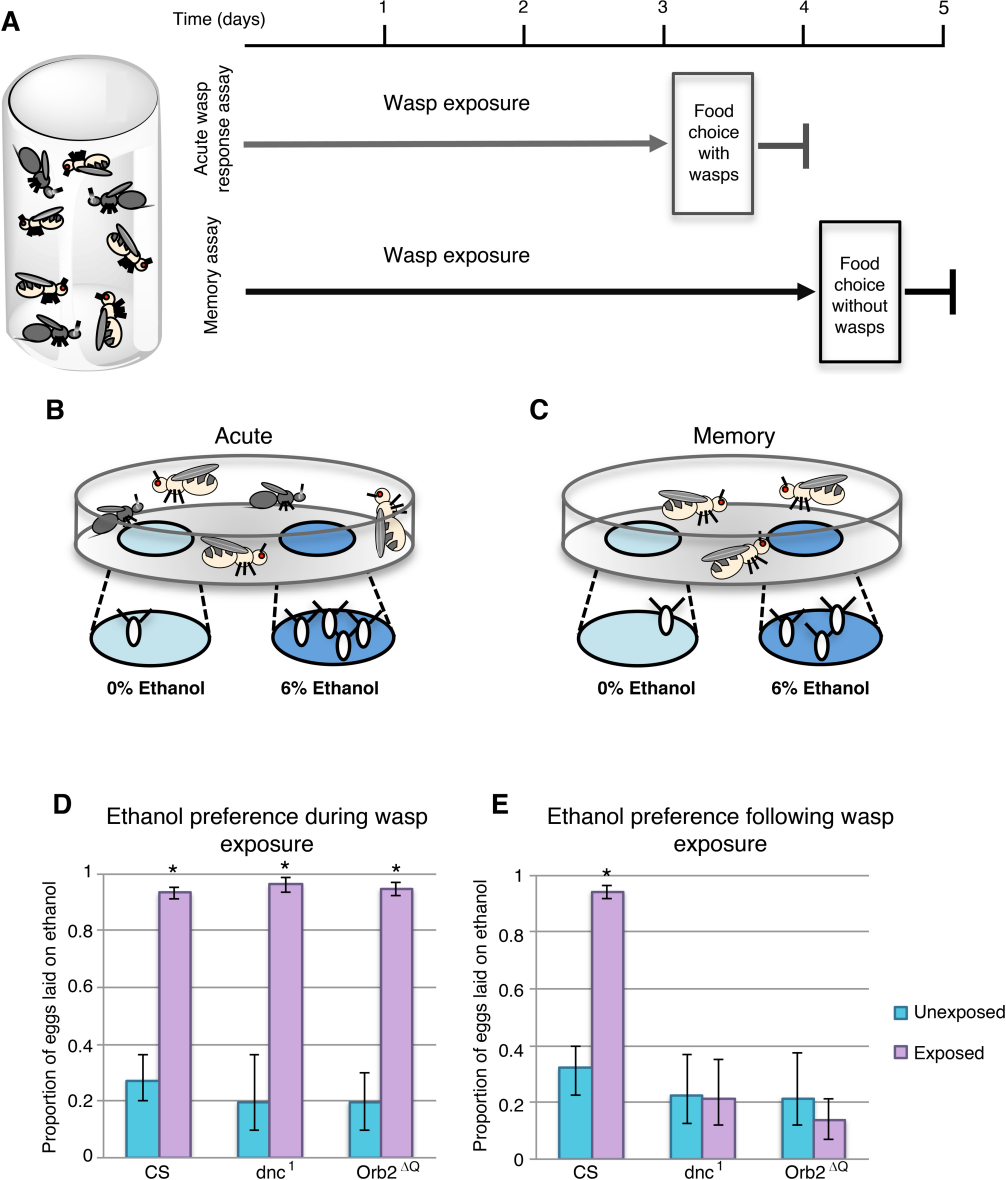
Wasp exposure causes ethanol seeking and long-term memory formation in fruit flies. Flies are cohabitated with wasps for either 3 days (acute assay) or 4 days (memory assay) (**A**). At the end of the exposure period flies are given two food options, ethanol and control food, either in the presence of wasps (**B**) or in the absence of wasps (**C**). CS flies as well as memory mutants have an acute wasp response where the ethanol food is preferred as an oviposition substrate (**D**). However, only CS flies maintain ethanol preference during the wasp memory assay (**E**). Error bars indicate bootstrap 95% confidence intervals.

It has been previously observed that when *Drosophila melanogaster* are cohabitated with the parasitic wasp *Leptopilina heterotoma* (LH14), flies will seek out ethanol containing food [29,30]. Further, when wasps are removed from the environment, female flies continue to favor ethanol-containing food as an oviposition substrate, a behavior that persists through the process of long-term memory formation. Using a similar experimental design we were able to replicate these previous findings (Fig 1A). Eggs were counted at the end of a 24-hour period, the proportion of eggs laid on ethanol food was calculated for each cage, giving an ethanol preference index; where a proportion of 0.5, or 50%, would indicate indifference to the presence of ethanol food. Under our environmental conditions, the baseline of ethanol preference in unexposed flies was approximately 20–30% (Fig 1D and 1E), signifying an avoidance of ethanol food. However, in the presence of wasps this ethanol preference increases to 93% (p-value 1.08E-5) in wild type Canton S (CS) flies (Fig 1D), illustrating a strong affinity for ethanol food under these conditions. In wild type flies, the ethanol preference is maintained following wasp exposure period, with female flies displaying an ethanol preference of 94% in the absence of wasps, which is significantly increased when compared to 32% unexposed (p-value 1.8E-4) (Fig 1E). These findings illustrate a behavioral switch perpetuated by the memory formed during wasp exposure. This ethanol seeking behavior has been shown to be robust across genetic backgrounds, and the maintenance of the ethanol seeking behavior is reliant on long-term memory formation. As expected, when we tested the classic memory mutants dnc^1^ and Orb2^ΔQ^, flies were able to respond to wasps, increasing from 19% and 19% to 96% and 94% ethanol preference respectively (p-values 1.5E-4, 1.7E-4). However, these mutants were not able to form memory, showing no significant increase in ethanol-seeking behavior after the wasp exposure period (p-values 0.96 dnc^1^, 0.42 Orb2^ΔQ^) (Fig 1D and 1E). These data support the previous finding that ethanol seeking post-wasp exposure is dependent on long-term memory formation.

### Gene expression

**Fig 2:**
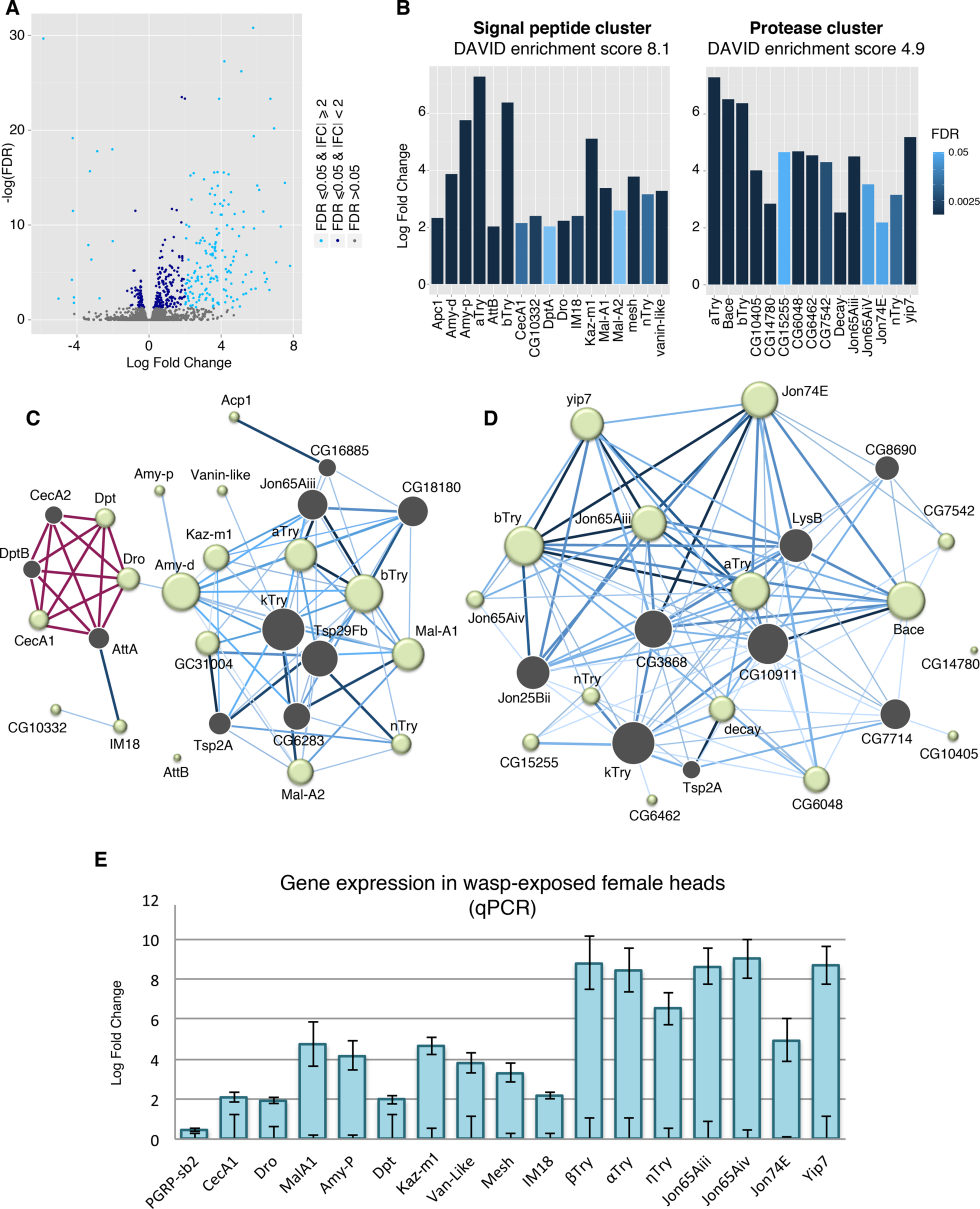
Signal peptide and protease genes are differentially regulated following wasp memory formation. Volcano plot displays sequencing results from wasp-exposed fly heads. Differentially regulated genes with FDR = < 0.05 are shown in dark blue points, genes with significant FDR and a log_2_ fold change magnitude of 2 or more are shown in light blue (**A**). Signal peptide and protease gene clusters were identified as enriched from a DAVID analysis. Bar shading indicates FDR, all genes shown have FDR = < 0.05 (**B**). IMP network analysis was preformed for the signal peptide cluster (**C**) and protease cluster (**D**) to generate a network of interactions amongst genes. In panels **C** and **D**, each node represents a gene; a green node indicates an input gene and grey node is a predicted interacting gene within the network, size of the node reflects the number of interactions. Known interactions are shown in red, blue edges are predicted interactions—the darker the edge the higher the predicted interaction score. A subset of genes identified from the sequencing was validated with qPCR. Samples were normalized to unexposed, the lower error bars represent SE of control group, upper error bars show SE of the exposed samples (**E**).

To explore gene expression changes that correspond to this long-term memory formation, we sequenced four-day wasp exposed and paired unexposed head samples. A total of 165 genes had at least a log_2_ fold increase of 2, and 14 genes were decreased by log_2_ fold of 2 or greater, with a false discovery rate (FDR) of 0.05 or less (Fig 2A, S1 Table). Of these differentially expressed genes, six functional gene clusters were identified as enriched in a DAVID analysis with enrichment greater than three. In order from highest to lowest enrichment clusters are as follows; chitin binding and extracellular region, signal peptides, attacin-related, proteases, glycosidase and sucrose metabolism, and defense response to fungus (S1 Fig, S2 Table).

Similar results were observed when the fold change restriction was removed, and all genes with significant FDR were used as input. This analysis identified five functional clusters with enrichment greater than three. In descending order, these clusters are annotated as chitin binding, signal peptides, DM9 domain proteins, sugar metabolism, and proteases (S3 Table).

The DAVID analysis generated from the gene list with both log2 fold change cutoff and significant FDR returned two groups of particular interest: A protease cluster with enrichment of 4.9, and a signal peptide cluster with enrichment of 8.1 (Fig 2B, S2 & S3 Figs). Given the potential biological relevance of these groups, genes from these clusters with a minimum log_2_ fold change of 2 were used to generate interaction networks through Integrative Multi-species Prediction (IMP). IMP predicts and graphically displays interaction between the genes as well as key genes predicted to be part of the network (Fig 2C and 2D). It is noteworthy that the trypsin genes are cross-listed in both protease and signal peptide clusters, and this fact is partly responsible for some overlap between the two interaction networks. It is reasonable to assume that these clusters are integrated into larger networks and pathways, and possibly interact with one another. One observation in support of this possibility is the presence of *Jon65Aiii*, a member of the protease cluster, as a predicted node of the signal peptide network. Additional genes with overlap between the clusters are the predicted node of *Tsp2A* as well as *κTry*. Of interest, *κTry* is the gene with the greatest number of interactions in both clusters totaling 13 edges in signal peptide network, and 18 edges in protease network. Overall, the protease cluster was more highly connected and this necessitated the use of more stringent prediction parameters for this cluster to focus the analysis. In this network, after *κTry* the genes with the greatest number of interactions are *CG10911* (17 edges), *CG3868* (15 edges), and *αTry* (14 edges) (Fig 2D).

The most connected genes in the signal peptide cluster are *Amy-d*, *βTry*, *Tsp2A* with 12 edges each (Fig 2C). A subset of genes identified by sequencing was further confirmed by quantitative polymerase chain reaction (qPCR) and validated as significantly differentially expressed (Fig 2E, S4 Table). *PGRP-sb2* was used as a negative control, as it was not identified as differentially regulated in the sequencing data. P-values for all tests can be found in S5 Table.

Classical memory genes were noticeably absent for the list of differentially expressed genes; many classical memory genes, such as *rut*, *dnc*, and *amn*, were identified through mutagenesis screens rather than differential gene expression [31–33]. In addition, more recent memory studies have not observed these traditional memory genes to be differentially expressed during memory formation on a global scale [34,35]. Therefore, the absence of these genes in our differentially expressed gene list is not surprising. However, two previously identified memory genes were present in the sequencing list with significant FDR: *RYa-R*, a neuropeptide receptor (log_2_ fold change 3.4, FDR 1.25e-7) and *scb*, a member of the integrin alpha chain family, (log_2_ fold change 0.57, FDR 1.37e-4) [36,37]. These findings function as a partial positive control within the data set.

### Dynamic gene expression across time

**Fig 3:**
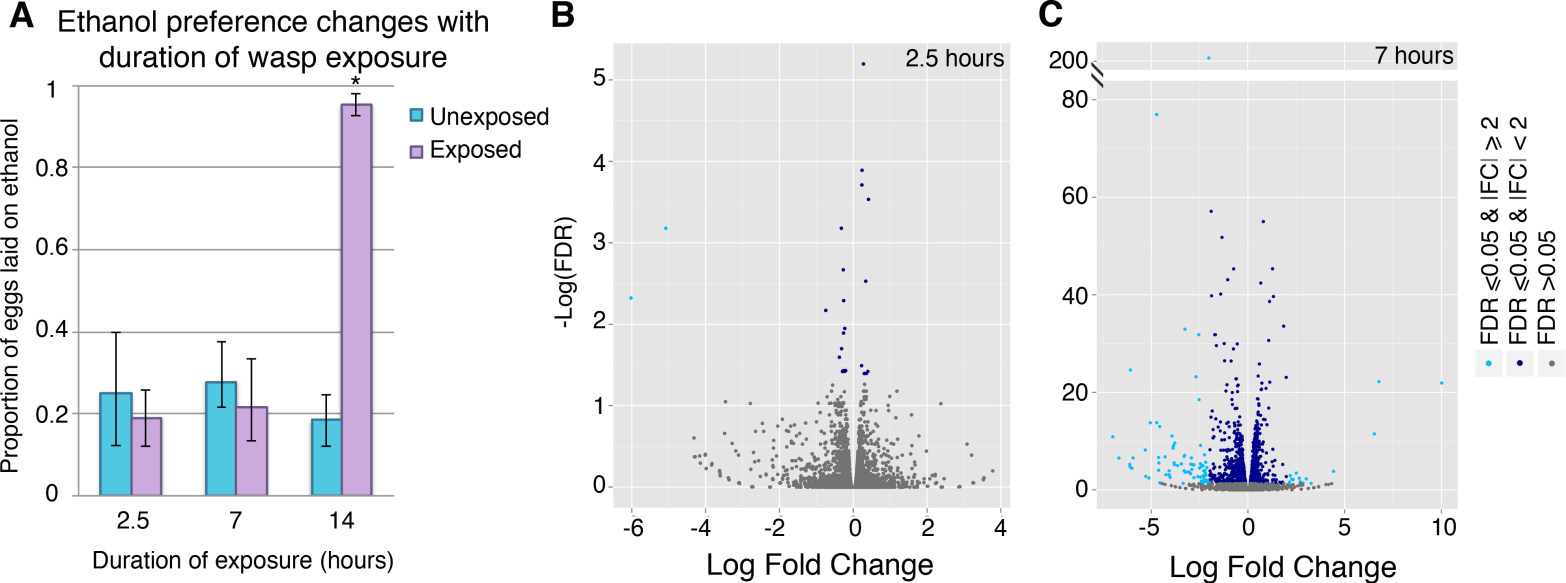
Differential gene expression occurs through the memory formation process. Ethanol preference depends on the length of wasp exposure, 2.5 and 7 hours of exposure shows no significant ethanol preference change (**A**). Volcano plots display sequencing results from heads of flies exposed to wasps for 2.5 hours (**B**) and 7 hours (**C**). Genes with significant FDR (= < 0.05) are shown in dark blue points, genes with significant FDR and a log_2_ fold change magnitude of 2 or greater are shown in light blue. Error bars indicate bootstrap 95% confidence intervals.

The process of memory formation is not instantaneous. In most learning paradigms, the animal requires repeated training sessions or extended duration of exposure to the stimulus for memory formation to occur. To capture transcriptional changes involved in the early stages of memory consolidation, we titrated the length of exposure to identify a wasp-exposure interval that did not confer the ethanol seeking behavior. Time points of 2.5, 7, and 14 hours were tested. We observed that neither 2.5 hours nor 7 hours of exposure is sufficient to trigger a behavioral switch in these flies. However, by 14 hours memory has been formed, as seen by the ethanol preference of 95% in the exposed group compared to 18% in the unexposed group (p-value 1.6E-4) (Fig 3A). Given these data, we hypothesized that unique memory-related genes not detected in the 4-day wasp-exposed samples, may be differentially regulated at these earlier time points.

**Fig 4:**
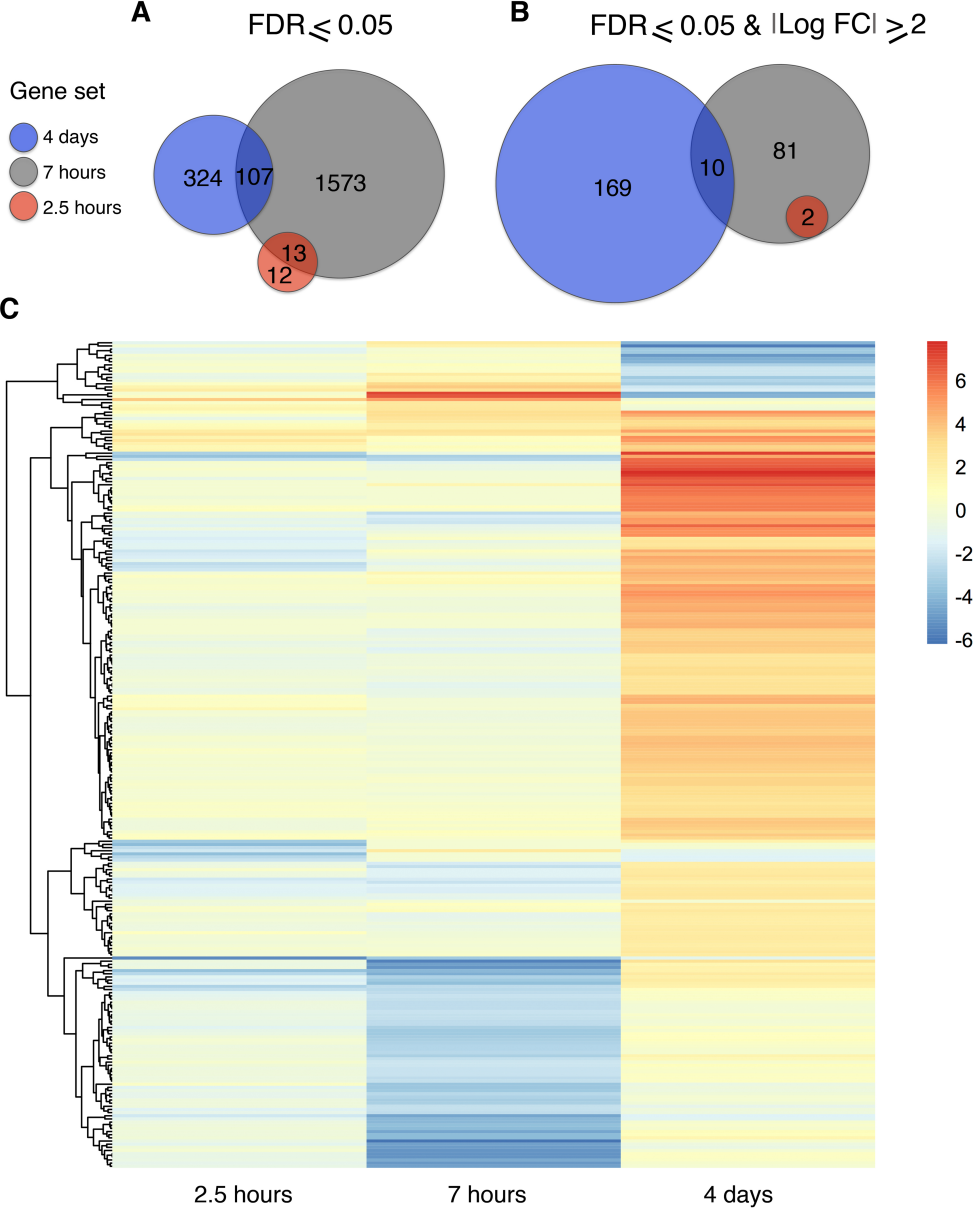
Gene expression is dynamic across memory formation time course. Differentially expressed gene totals are shown for each time point 4 days (blue) 7 hours (grey) 2.5 hours (red). Genes with significant FDR are displayed as a Venn diagram (**A**). Alternatively, genes with significant FDR and minimum log_2_ fold change magnitude of 2 are shown with overlap (**B**). Heat map illustrates gene expression across time, blue indicating down regulation and red up regulation of transcript (**C**). All genes from panel B are included in the heat map.

To explore this possibility, sequencing data was generated from female fly heads with 2.5 and 7 hours of exposure (Fig 3B and 3C). The 2.5-hour time point revealed minimal differential gene expression (Fig 3B, Fig 4A and 4B). Two genes, *Bsg25A* and *Elba3*, were identified as having both a log_2_ fold change magnitude of 2 and significant FDR (FDR = <0.05). No genes with significant FDR were identified as overlapping with the 4-day differentially expressed gene list (Fig 4A).

**Fig 5:**
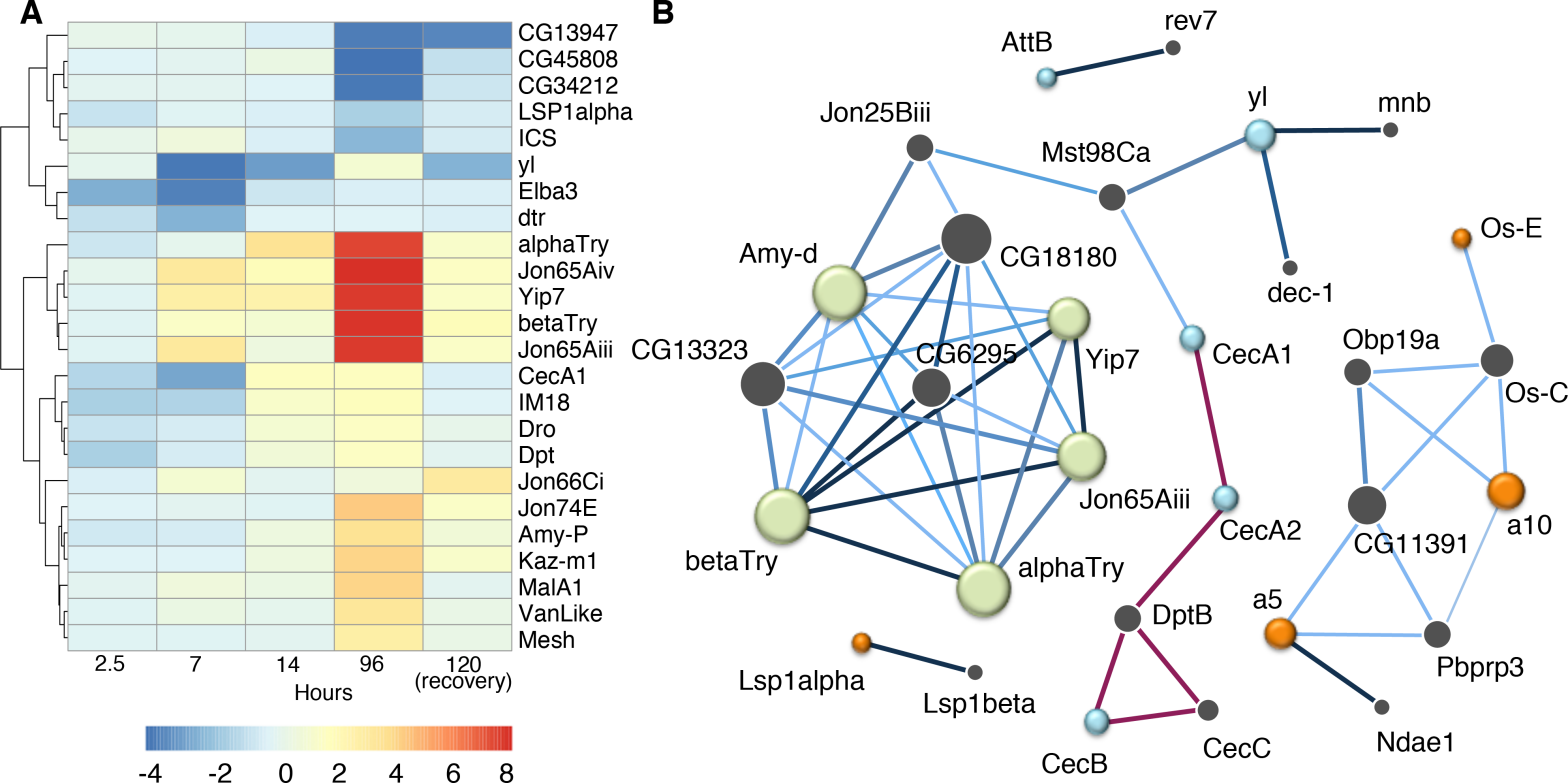
Temporal gene expression points to interacting networks during memory formation. Heat map illustrates gene expression as quantified by qPCR following 2.5, 7, and 96 hours of wasp exposure. A fourth time point, 24 hours of recovery following wasp exposure, was included in the cluster analysis; shading indicates direction and magnitude of gene expression change (**A**). IMP network analysis was preformed for differentially regulated signal peptide genes from the 7-hour time point and a subset of genes from the 4-day signal peptide cluster (**B**). Each node represents a gene, a green node indicates a gene from the 4-day signal peptide cluster. Orange and blue nodes are genes from the 7-hour signal peptide cluster, color indicates that the gene is up or down regulated (orange and blue respectively). Grey nodes represent predicted interacting genes within the network, size of the node reflects the number of interactions. Known interactions are shown in red, blue edges are predicted interactions the darker the edge the higher the predicted interaction score.

At the 7-hour time point, 1693 genes were differentially expressed with a significant FDR, 93 of which had a minimum fold change magnitude of 4. Down regulated genes constituted the bulk of this gene list, with 79 genes down regulated compared to only 14 genes up regulated (S6 Table). When compared to the 4-day sequencing data, 10 genes were found to overlap between the two gene sets. Of these shared genes, only two genes are differentially regulated in the same direction; *Jon65Aiii* and *Jon65Aiv* are both up regulated (Fig 4C, Fig 5A, S7 Table). Interestingly, *Bsg25A* and *Elba3* are also down regulated at the 7-hour time point, resulting in complete overlap of the 2.5-hour gene list (Fig 4B).

We further examined the temporal dynamics of gene expression by measuring RNA levels following a 14-hour wasp exposure (Fig 5, S4 Table). Considerable overlap exists between the 4-day and 14-hour time point: For instance, *αTry*, *βTry*, *yip7*, *Jon65AiV*, and *Jon65Aiii* were up-regulated at 14 hours, but to a lesser degree than the 4-day time point. Alternatively, *IM18* and *CecA1* had similar expression levels at the two time points. These differences in gene expression patterns hint at multiple regulatory pathways governing gene expression. Certain genes may reach their maximum induction quickly, or have their mRNA strictly regulated, resulting in plateaued RNA levels between the 14-hour and 4-day time points. Other genes may maintain increasing mRNA production as there is continuing memory formation or prolonged exposure to stress conditions.

In addition to dynamics of a specific gene across time, gene-gene interactions may be key to the memory formation process as well. Although the gene sets show limited overlap in differentially expressed genes, it is noteworthy that signal peptides were found to be enriched in a DAVID analysis of the 7-hour data set (S8 Table). Many of these genes are different from those in the 4-day signal peptide cluster; however, IMP network prediction suggests possible interactions between genes in the two clusters (Fig 5B). Further, the network analysis predicted nodes within the 4-day signal peptide cluster that are differentially regulated in the 7-hour samples. In other words, genes that are predicted to interact with the 4-day signal peptide cluster are differentially regulated at an earlier time. Specifically, *CecA1*, *Dpt* and *Dro* are up regulated in the 4-day exposed samples; these genes are known to interact with *CecA2*, *DptB*, and *AttA* (Fig 2C). These three predicted nodes of *CecA2*, *DptB*, and *AttA* are differentially regulated with significant FDR, although only *CecA2* meets the log_2_ fold change threshold; with log_2_ fold change values of -2.86, -1.3, and -1.85 respectively.

To explore the role of these genes following memory formation, we measured transcript abundance in flies after a 24-hour recovery period following wasp removal. Although several genes exhibited a trend towards up or down regulation, none of the genes assayed had statistically significant differential gene expression, likely due to the unusually high variance in these samples (Fig 5A, S4 Table).

### Behavioral validation

**Fig 6:**
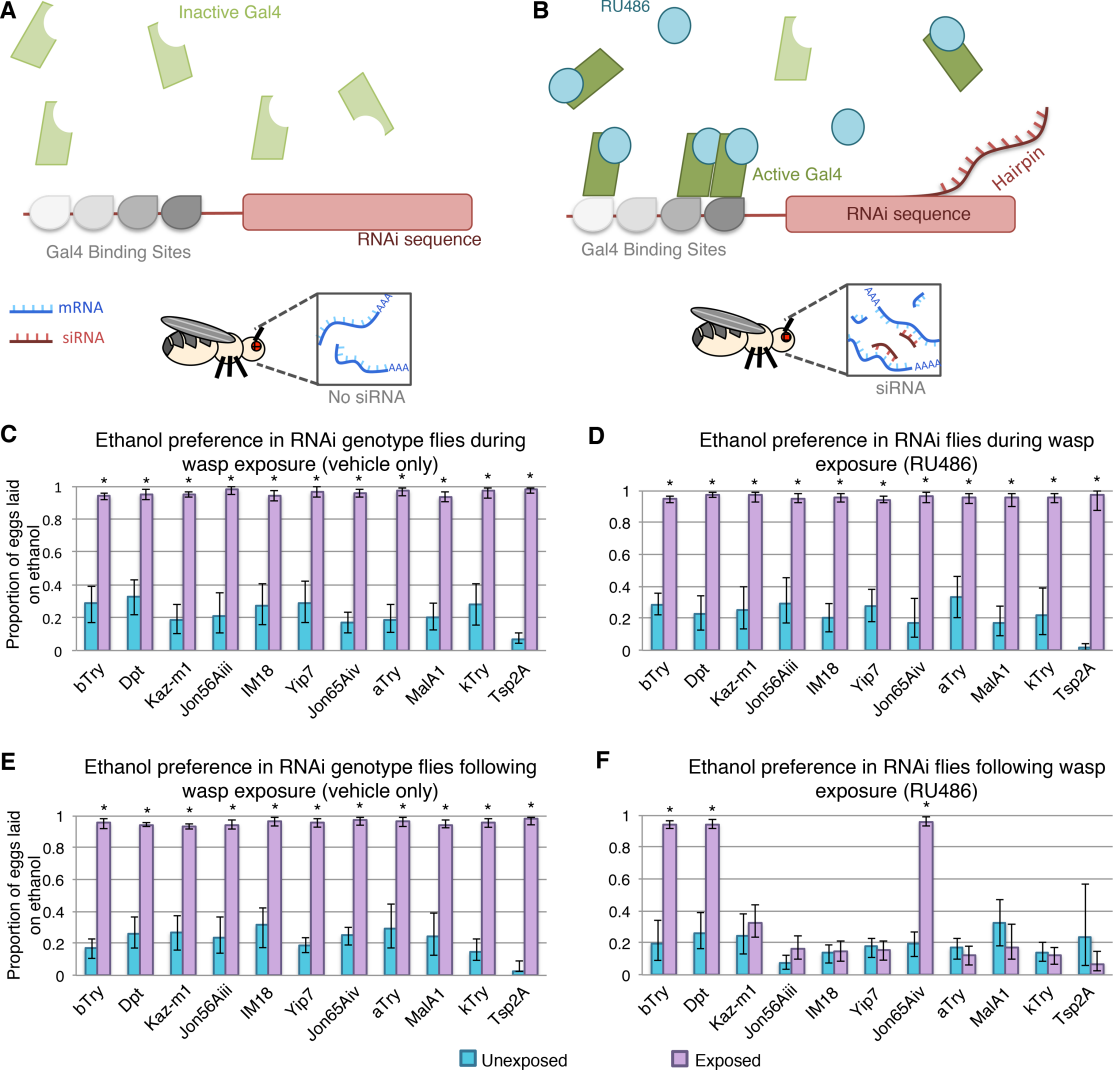
Conditional knockdown in neurons identifies important genes for memory formation. Gal4-Switch system expresses inactive Gal4 transcription factor, resulting in no RNAi hairpin (**A**). When flies are fed RU486, the Gal4-switch becomes active and can bind DNA, driving RNAi expression (**B**). Acute wasp exposure experiment shows that flies have ethanol seeking behavior when fed vehicle (**C**) or RU486 (**D**). All genotypes maintain ethanol seeking following wasp exposure when treated with vehicle only (**E**). Ethanol seeking in the memory assay is disrupted for flies expressing RNAi to a subset of genes (**F**). Error bars indicate bootstrap 95% confidence intervals.

To evaluate the functional significance of the differentially expressed genes and their possible role in memory formation, we conducted behavioral experiments paired with gene knock down. Initial experiments were preformed with the Elav-Gal4 switch driver, an inducible pan neuronal driver, with the specific advantage of the Gal4 transcription factor being active only when the RU486 ligand is present. In this system the RU486 must be externally provided, thus allowing for temporal control of the RNAi expression (Fig 6A and 6B). Elav-Gal4 switch lines were crossed to UAS-RNAi lines for a subset of differentially regulated genes; *βTry*, *Dpt*, *Kaz-M1*, *Jon65Aiii*, *IM18*, *yip7*, *Jon65Aiv*, *αTry*, *MalA1*. *κTry* and *Tsp2A* were additionally tested as both were predicted to be an interacting gene in the IMP analysis although not differentially expressed. Expression level of *κTry* was confirmed with qPCR as not differentially expressed following wasp exposure (S4 Table). All of these genotypes had significant ethanol preference in the acute assays for vehicle only as well as with RU486 feeding, indicating that vehicle or RNAi depletion of each gene did not cause any defects in animals' ability to perceive and respond to the presence of the wasp predator (Fig 6C and 6D). All 10 genotypes had memory formation following wasp exposure with vehicle only feeding, indicating that ingestion of vehicle (5% methanol) did not disrupt memory formation (Fig 6E). However, after wasp removal *Kaz-M1*, *Jon65Aiii*, *IM18*, *yip7*, *αTry*, *MalA1*, *κTry* and *Tsp2A* displayed memory defects upon RNAi knock down (Fig 6F). These data indicate that each genotype is able to respond to wasps and form memory when the RNA-hairpin is not expressed. In addition, these results show that the RNA-hairpin expression does not inhibit the ethanol-seeking wasp response in the presence of wasps, but rather after wasp removal, RNAi depletion of certain genes interrupts memory formation or maintenance. Several attractive candidate genes did not result in memory defects, and we offer two conceivable explanations beyond the hypothesis that they are not relevant memory genes. First, it is possible that we had insufficient knock down of the gene to illicit a phenotype. Secondly, it is also reasonable to consider that, as the sequencing was performed on whole heads, some of the differentially expressed genes may be in non-neuronal tissues. In such a case our pan-neuronal driver would not express the RNA-hairpin in the relevant cell type.

**Fig 7:**
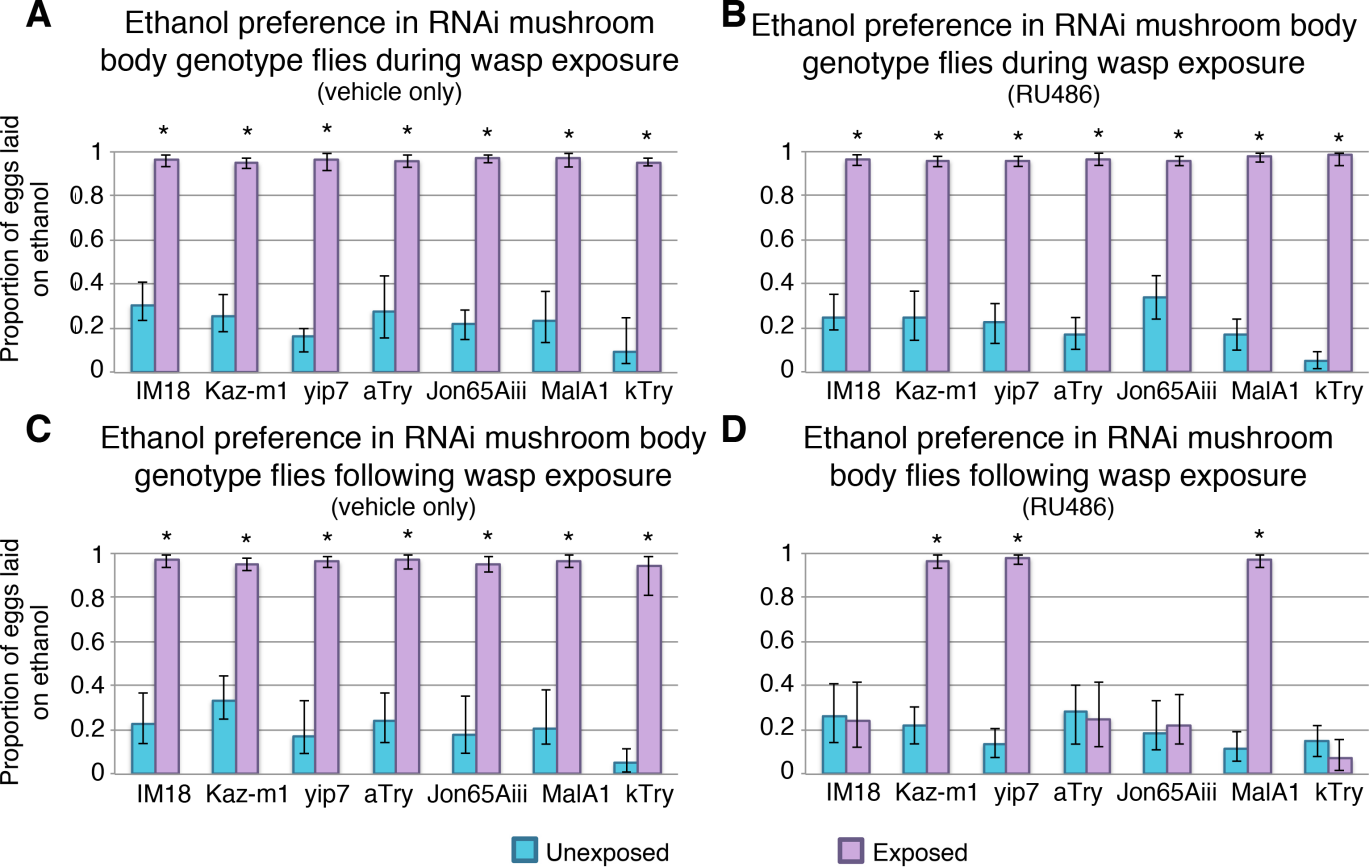
Conditional knockdown in mushroom body neurons identifies genes acting specifically within the learning and memory center of the brain. Flies have an ethanol seeking behavior in the presence of wasps during vehicle feeding (**A**) and RU486 feeding (**B**). Ethanol preference is maintained following wasp removal when flies are treated with vehicle only (**C**). Ethanol seeking is not maintained for all RNAi lines when fed RU486, indicating disruption in memory formation (**D**). Error bars indicate bootstrap 95% confidence intervals.

**Fig 8:**
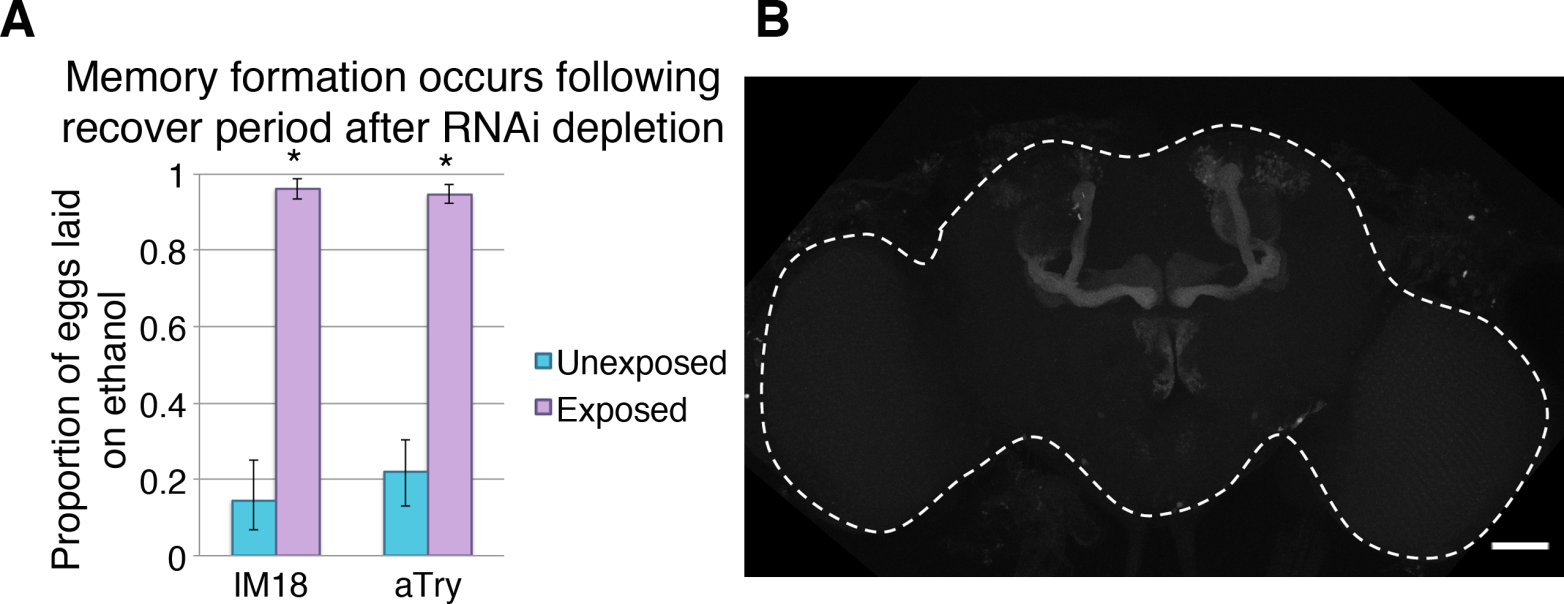
Conditional knockdown in mushroom body does not permanently inhibit memory formation. Flies treated with RU486 that are transitioned back to normal food are able to form memory following recovery period (**A**). Mushroom body neurons expressing GFP under the MB switch inducible driver, dashed white line outlines the brain (**B**).

To understand where in the nervous system these genes function, we used a more specific Gal4 driver line, which expresses in the learning and memory center of the *Drosophila* brain, known as the mushroom body (MB). Genes that yielded memory defects in the previous experiments were tested with this more specific MB-Gal4 switch driver. All genotypes tested had ethanol seeking behavior in the presence of wasps (Fig 7A and 7B). Additionally, memory formation of the genotypes was not disabled when treated with vehicle only (Fig 7C). However, *IM18*, *Jon65Aiii*, *αTry*, and *ĸTry* knock down in the MB caused memory defects. We considered the possibility that RNAi depletion of any essential gene in MB neurons could damage or kill cells important for memory consolidation or maintenance. In this scenario such genes would not be essential for memory *per se*, but instead they could simply be required for cell survival or neuronal activity. To test this possibility flies were treated with RU486 to induce RNAi depletion, as preformed previously, and then allowed to ‘recover’ for 4 days without RU486 feeding. Subsequently, they were tested for memory formation after exposure to predatory wasp. We found that RNAi knock down of IM18 and alpha Try before wasp exposure did not lead to disruption of memory (Fig 8). These observations suggest that knock down of these genes does not permanently damage the neurons of the mushroom body, which would prevent memory formation as an indirect consequence of gross neurological defect. Therefore, we conclude that *IM18* and *αTry* are likely to be required for memory formation or maintenance as opposed to indirectly disrupting memory by affecting general neuronal processes and survival.

Of the validated genes resulting in memory defects in the signal peptide annotation cluster, little is known about functional processes, making speculation about their mechanistic role in the brain challenging. For instance, *IM18* has no identified protein similarity nor does it have a described function [38]. *Kaz-m1* has been studied in greater detail, located in the E(spl)-C locus it is a Kazal family protease inhibitor [39]: Yet, basic information on binding partners and localization remain unknown, making specific predictions impractical. However, general trends may point to larger scale pathways and processes. Jak/Stat signaling has been observed in the Kenyon cells of the mushroom body, leading to the hypotheses that immune signaling is triggering actin cytoskeleton arrangement or chromatin remodeling as part of memory formation [40]. Alternatively, it is possible that the activation of immune pathways is indicative of synaptic pruning similar to what is observed during nervous system development [41].

The protease annotation cluster has few well-characterized genes, but predicted functions of these genes and expression patterns provide useful information nonetheless. Yip7 RNA expression has been observed in Drosophila surface glia, although its role in these cells remains unknown [42]. More generally, serine proteases are suggested to interact with protease activated receptors. Further, Trypsin-like proteins have been observed at presynaptic terminals in mammalian models, leading to speculation that such genes are involved in synaptic plasticity and long term potentiation [43,44]. It is possible that similar processes are occurring in the Drosophila nervous system and that the up regulation of these genes during memory formation is part of synaptic remodeling.

## Discussion

Memory formation, maintenance, and retrieval occur through an intricate system where information from new sensory inputs and existing neurocircuitry is consolidated. This process is massively complex and to date we lack a clear, global understanding of it. In the case of LTM, memory formation relies on basic functions, such as gene expression and protein synthesis. Dissecting these mechanisms and their dynamics brings the field closer to this large-scale view of learning and memory. In this study we approached the question of gene expression dynamics during memory formation in a novel non-associative LTM paradigm. We confirm previous findings that wasp exposure triggers a LTM dependent behavioral change resulting in female flies preferring ethanol-containing food as an oviposition substrate; and report RNA sequencing data specific to this wasp induced LTM.

It is well established that dynamic gene expression is necessary for persistent memory [2]. In this sense the results presented in this paper may not be surprising, where we identified 179 genes that were differentially regulated following a four day exposure to wasps. It is noteworthy, however, that more than 90% of these genes were up regulated. Previous gene expression studies have shown conflicting results in this regard. The distribution of differentially expressed genes is varied and seems to depend on the neuron type, paradigm, and timing of collection [18,21,45]. Given that samples were collected immediately following the removal of the wasps, it is possible that a number of these genes are activity regulated genes (ARGs), which are typically up regulated following neuronal firing [16,17]; although our sequencing data from earlier time points does not provide strong support for this hypothesis. Alternatively, this observation may be a behavioral paradigm specific phenomenon, possibly unique to non-associative learning.

Based on this data we identified six enriched functional gene clusters from our DAVID analysis. The gene cluster with highest enrichment related to chitin binding. A previous study has implicated peptidoglycans in the behavioral changes resulting from bacterial infection [46]. Enrichment of genes with these functional annotations may hint at a similar role for them in the modification of other defense related behaviors. In addition to this, two functional clusters, signal peptides and proteases, were of particular interest based on biological inference. These experiments have therefore generated a substantial candidate list of novel up regulated genes, some of which may be important for memory.

The signal peptide cluster was of particular interest, as it contained a number of immune associated genes. In particular, immune deficiency pathway (IMD) components appeared in the sequencing, in addition to two immune inducible genes *IM18* and *AttB*. The IMD genes identified included *CecA1*, *CecA2*, *Dro*, *AttA*, *Dpt*, and *DptB* and were differentially regulated at various time points. These findings raise questions about the role of immune genes in learning and memory. The immune system in Drosophila has long been linked to inflammation and neurodegeneration [47–50]: Yet this would be a surprising discovery in a LTM paradigm. We suggest that a more likely scenario relates to recent studies that have been revealing a larger role for immune peptides in the nervous system, such as their participation in neuronal differentiation [51–53]. It is becoming clear that immune genes, and the IMD pathway, have non-canonical functions in the nervous system. Sleep regulation is one key area in which these genes are being examined. In particular *Dro* and *AttB* increase in expression with sleep deprivation, and more generally IMD genes are involved in sleep regulation [54,55]. Given the profound effect of sleep on learning and memory, we speculate that these immune genes are contributing to neuronal function in some way. Another tantalizing observation is that a *STAT92E* isoform is up regulated following courtship rejection training, perhaps implicating immune regulatory networks outside of the IMD pathway [35]. The precise mechanism underlying the importance of immune genes in the brain currently remains unclear. However, it has been speculated that perhaps immune signaling pathways are used as communication between neuron and non-neuronal tissues, such as the fat body [55,56]. Additional hypotheses have been put forth that focus on post-transcriptional actions, for instance NF-ĸB, the upstream activator of several immune pathways, has been implicated in affecting receptor density and synaptic stability [57,58].

Although this and other papers have presented plausible evidence for the role of immune genes in neurons; it is nonetheless important to consider indirect, system wide effects of the immune system and related processes. It has been shown that long term memory formation is energy demanding, as it requires protein synthesis and synaptic remodeling. These energy demands have measurable phenotypic outcomes, for instance one study found that Drosophila with enhanced memory in turn had reduced resiliency to starvation and dehydration [59]. Conversely, fruit flies under starvation conditions display impaired memory [60]. Such findings reinforce the notion that neurological processes such as long-term memory formation require tradeoffs. We would therefore be remiss to not consider the energy demands of the immune system. It is possible that these genes are negative regulators of the immune system, and by up-regulating these genes, energy is redirected from immunity to the brain. Alternatively, such genes may be protective against oxidative and immune related stress damage on the nervous system. Given the complexity of memory formation at the organism level, such hypotheses will need to be rigorously addressed in future works.

The second gene cluster explored was the protease enrichment group. Two genes identified showed particularly interesting trend: *Jon65Aiii* and *Jon65AiV* were the only two genes up-regulated at both the 7-hour and four-day time points. Their increased gene expression at 7 hours, before memory formation has occurred may indicate a role for them in memory initiation or possibly unidentified ARGs. Of further note, *Jon65Aiii* is cross-listed between the predicted gene networks for the signal peptide and protease clusters. Although not experimentally verified, the overlap between networks illustrates the complex interactions that may be at play in LTM. The predicted gene nodes of *κTry* and *Tsp2A*, again shared between both networks, add additional emphasis to complex interactions between gene networks.

Of the candidate genes, several were experimentally validated as LTM genes. Using a pan-neuronal conditional RNAi, we were able to show that *Kaz-M1*, *Jon65Aii*, *IM18*, *yip7*, *αTry*, and *MalA1* are essential genes for this memory paradigm. In addition, we used network prediction to identify genes possibly important to the memory formation process that are not differentially regulated in our data sets. This computational method produced *κTry* and *Tsp2A* as candidate memory genes; remarkably, RNAi knock down of these genes yielded a memory phenotype, further validating such predictive tools. It is important to emphasize that the induction of double-stranded RNA and RNAi depletion of any of these genes did not inhibit wasp perception or the behavioral response; instead the memory formation itself was interrupted.

Additional nuances are being introduced to the field of study as the distinctions between LTM and memory consolidation are established [61]. The reinforcement and establishment of a memory outside of the traditional learning and memory centers of the brain, such as the hippocampus in mammalian systems, may be viewed as fundamentally different from LTM [62,63]. Inhibition of certain waves of protein synthesis appears to affect memory persistence, instead of memory formation, in some model systems [5,7]. However, the line separating these two processes is not well defined and the role of previously identified LTM genes may need to be evaluated in the memory consolidation process to truly understand their functional significance. Of the seven genes tested, *IM18*, *αTry*, *Jon65Aiii*, and *κTry* showed memory defects, indicating that these genes have essential functions in the learning and memory center of the Drosophila brain.

Identifying genes acting specifically within the MB was initially unexpected, as the expression data was generated from whole heads. It is possible that these genes have multiple functions within the brain, or possibly that they are highly expressed in enough cells to overcome this whole head dilution. Also surprising was the lack of differential gene expression at 2.5 hours. Previous data would suggest that ARGs should be activated within this time window. However, data also suggests that the shared number of ARGs across neuron type is limited, this may restrict the genes found due to the pooling effect caused by full head samples [19]. Future approaches that can detect single cell changes in gene expression will be particularly useful in spatially mapping how and where changes in gene regulation contribute to learning and memory.

Overall, this study has identified novel genes involved in non-associative LTM, and we have attempted to place these genes into a larger network context. Three of the genes identified have critical roles in the MB, while the remaining memory genes are likely acting either up stream or down stream of the learning and memory center. Although the behavioral experimentation suggests important roles for these genes, their specific functions are not known and require additional experimentation to elucidate mechanism. These data also support the existing literature that point to the IMD pathway as an important player in learning and memory. With continued research and the use of bioinformatics tools we hope these data complement and inform future studies into the process of LTM formation, and in combination with a robust non-associative learning and memory approach, we propose that gene function can be further dissected into learning, memory consolidation, and memory maintenance activities.

## Methods

### Fly husbandry and behavioral experiments

Stocks were maintained on standard Drosophila cornmeal-molasses media at room temperature (S9 Table). Memory formation experiments were conducted in vials (9.5 by 2.5 cm) containing 40 female flies and 10 male flies. The wasp-exposed group had the addition of 20 female Lh14 wasps, as previously described [30]. Wasp exposures were maintained for 2.5, 7, 14, and 96 hours (4 days). Memory formation was determined by ethanol preference, measured by a food choice assay. Briefly, one male and five female flies were placed into cages with two food sources, one with 0% ethanol and the other 6% ethanol food. Flies remain in the cages for 24 hours, at which point the food plates are collected and the number of eggs laid on each food source is counted in a blinded manner, such that the counter is not aware of genotype or treatment. As noted previously, the baseline ethanol preference/avoidance is sensitive to both temperature and humidity; all experiments reported were performed in a room with over head lighting and maintained at 25° with 30% humidity. All memory experiments measured ethanol-seeking behavior immediately following the removal of the wasps.

Acute response experiments were conducted to determine the ability of the flies to respond to wasps irrespective of memory formation. These experiments were completed in similar fashion to the memory experiments, but with the addition of three female wasps in the cages of the exposed group during the food choice assay.

Knock down experiments were performed using the UAS-Gal4 Switch system, where the Gal4 transcription factor becomes active only in the presence of RU486 [64,65]. Instant food impregnated with either the drug or vehicle only was used as the delivery system for this method of genetic manipulation. Two grams of instant food was hydrated with 8 mL of either RU486 (0.22 mg/mL) in 5% methanol, or vehicle only (5% methanol). Flies were transferred to new food each day. Memory experiments consisted of four days of feeding concurrent with wasp exposure. Ethanol choice assays immediately followed the removal of wasp and RU486/methanol food. Alternatively, the acute response experiments were comprised of three days of feeding concurrent with wasp exposure, followed by the acute ethanol choice assay. In these acute response experiments the feeding protocol of drug or vehicle only was maintained during the ethanol choice assay.

For the above-mentioned experiments, each is comprised of 10 cages per group unless otherwise noted. The data presented from these experiments is shown as a proportion of eggs laid on ethanol food compared to the total egg number from the cage and plotted as an average of the 10 replicates. Error bars were generated through bootstrapping the mean with 95% confidence. P-values were calculated from the Mann-Whitney U test. All statistics were calculated in R (version 3.0.2 “Frisbee Sailing”), p-values for all tests can be found in S5 Table.

### RNA isolation and sequencing

Female flies were collected in 15 mL conical tubes, frozen in liquid nitrogen and briefly vortexed. Heads were separated using stackable steel sieves with pore size 710, 425, and 125 μm: Approximately 100 heads were collected for each replicate. Samples were maintained in Trizol at -80° until RNA isolation was performed using the miRNeasy Kit (Qiagen) with on-column DNase treatment. Four samples of each group were sent for sequencing on the illumina platform. Samples underwent rRNA reduction followed by random priming and were sequenced with a depth of 40 million reads. The adapters of short reads were trimmed by trimmomatic (version 0.33) [66]. The short reads were then mapped to *Drosophila melanogaster* reference genome (release 6.02) using STAR [67]. PCR duplicated short reads were removed by samtools (version 0.1.19) [68]. Read counts per gene were calculated via bedtools [69]. The differentially expressed gene analysis was performed with edgeR [70,71]. Expression values for the four-day wasp exposure experiment were calculated by normalizing the exposed group to the paired control samples. Additional time course sequencing experiments for hours 0, 2.5, and 7; expression values were determined by comparing to the 0 hour time point samples. Heat maps were generated using hierarchical clustering and the R package pheatmap. The sequencing heat map included every gene that had a log_2_ fold change magnitude of 2 or more and significant FDR at any single time point.

### DAVID analysis & Integrative Multi-species Prediction (IMP)

Genes with significant FDR (FDR = < 0.05) and log_2_ fold change of 1 were used in the primary DAVID enrichment analysis [72,73]. Generation of the graphical illustration of the DAVID network required integration of the gene clusters and the corresponding fold changes from edgeR using a customized Perl script. The DAVID plot was subsequently created using a customized R script using package plotrix (Version 3.6–1) and iGraph (Version 1.0.1) with a Fruchterman and Reingold layout [74,75]. This code can be found at https://github.com/chenhao392/flyMemoryProject. Secondary DAVID enrichment analysis was conducted using all genes with significant FDR (FDR = <0.05) regardless of the fold change value.

Genes from these groups with at least log_2_ fold change of 2 were used to generate interaction networks in IMP (http://imp.princeton.edu) [76]. The clusters identified by DAVID were analyzed independently. The signal peptide cluster analysis used a 0.13 minimum prediction threshold and 10 additional gene nodes limit for the generation of the network. Given the increased number of interactions within the protease cluster, a more stringent threshold of 0.2 minimum prediction score and 8 additional gene nodes limit.

The network analysis for both the 7-hour and 4-day signal peptide clusters used all 9 genes from the 7-hour time-point with at least a log_2_ fold change of 2. The genes Amy-P and Yip7 from the 4-day cluster we used as input genes in the analysis; other genes from this cluster were identified by IMP as predicted interacting genes.

### qPCR

cDNA was generated from RNA samples using the QuantiTect Reverse Transcription Kit (Qiagen). The iTaq Universal SYBR Green (BioRad) was used for the PCR reaction, and all primers were validated with standard curve before use (S10 Table). Gene specific data was normalized to actin and log_2_ fold change was calculated using the delta-delta CT method. An additional time point was collected post-memory formation: Following the 4 days of exposure, flies were separated from wasps and allowed to recover for 24 hours in a new vial before collection. All other time-points used RNA from the sequencing samples. Significance was determined by a two-tailed t-test. Statistical calculations were preformed in R (version 3.0.2 “Frisbee Sailing”).

## Supporting information

S1 Fig Differentially expressed genes form functional clusters in DAVID analysis. The DAVID network plot illustrates the many-to-many relationship between up-regulated genes and their enriched functional annotations. Up-regulated genes are denoted with a solid red-blue node. Edges connect these genes to their enriched functional annotations (grey nodes). The color of the gene node, ranging from blue to red, indicates the log fold change of the gene based on sequencing data.
(TIF)

S2 Fig Protease gene cluster within the DAVID enrichment analysis. Inset illustrates the entire DAVID enrichment network, doted box indicates region of magnification. Up regulated genes (colored nodes) connect to corresponding enriched functional annotations (grey nodes).
(TIF)

S3 Fig Magnification of functional clusters, including the signal peptide group within the DAVID analysis. Inset illustrates the entire DAVID enrichment network, doted box indicates region of magnification. Up regulated genes (colored nodes) connect to corresponding enriched functional annotations (grey nodes).
(TIF)

S1 Table Differentially expressed genes in female fly heads following 4 days of wasp exposure. Genes listed have and FDR = < 0.05 and minimum log_2_ fold change magnitude of 2.
(XLSX)

S2 Table Results of functional annotation clusters with DAVID analysis. Gene list used had a log2 fold change minimum of 1 and FDR less than 0.05. Enrichment score is indicated in parentheses with the cluster identification.
(XLSX)

S3 Table Results of functional annotation clusters with DAVID analysis. Gene list was generated with a FDR restriction of less than 0.05; no fold change minimum was used. Enrichment score is indicated in parentheses with the cluster identification.
(XLSX)

S4 Table Gene expression values as determined by qPCR across memory formation time points. Values represent average log_2_ fold change +/- SE.
(XLSX)

S5 Table P-values and statistical tests for all data comparisons. (XLSX)

S6 Table Differentially expressed genes in female fly heads following 7-hour wasp exposure. Genes listed have and FDR = < 0.05 and minimum log_2_ fold change magnitude of 2.
(XLSX)

S7 Table log_2_ fold change values for differentially expressed genes shared through time point gene sets. (XLSX)

S8 Table List of signal peptides identified in the 7-hour sequencing data set. (XLSX)

S9 Table Stock listing and genotypes of fly lines used in experiments. (XLSX)

S10 Table Sequence information for primers used in qPCR experiments. (XLSX)
