# Supplementary figures and images for: A systems level approach to temporal expression dynamics in Drosophila reveals clusters of long term memory genes
Source: PLoS Genet. 2017 Oct 30;13(10):e1007054. doi: 10.1371/journal.pgen.1007054 (PMC5679645; doi:10.1371/journal.pgen.1007054)

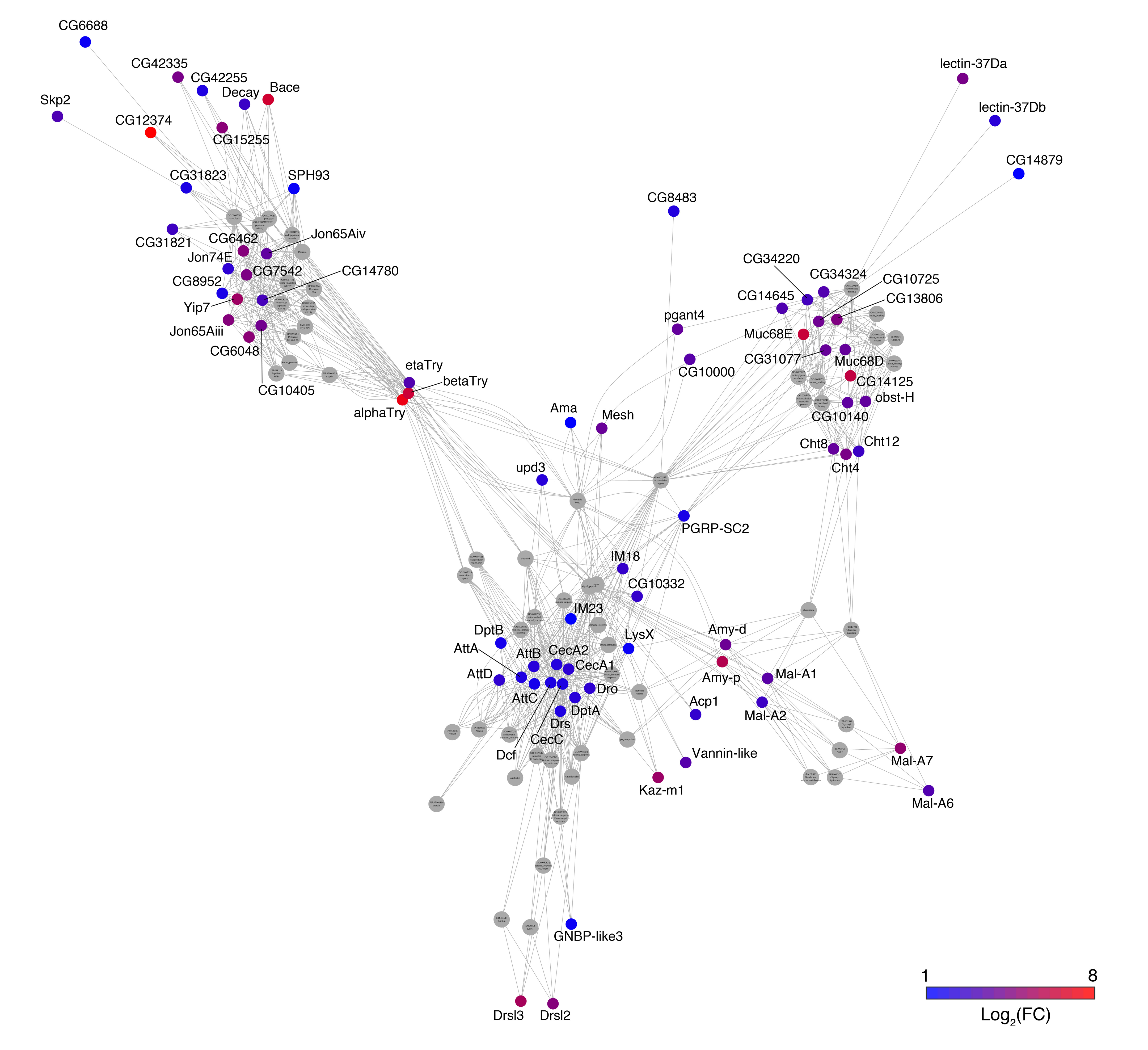

Supplement: S1 Fig — The DAVID network plot illustrates the many-to-many relationship between up-regulated genes and their enriched functional annotations. Up-regulated genes are denoted with a solid red-blue node. Edges connect these genes to their enriched functional annotations (grey nodes). The color of the gene node, ranging from blue to red, indicates the log fold change of the gene based on sequencing data. (TIF) [file pgen.1007054.s001.tif]

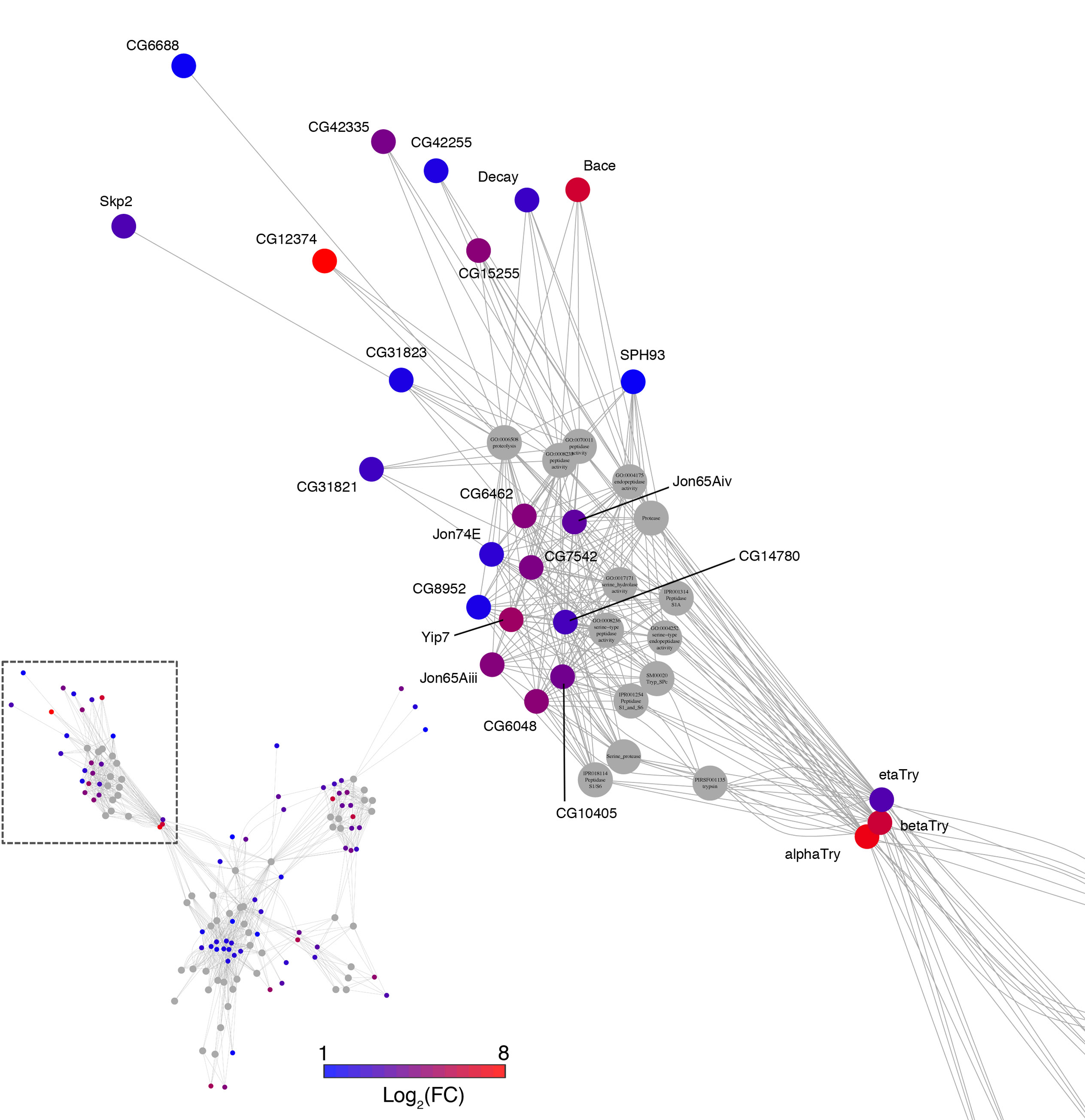

Supplement: S2 Fig — Inset illustrates the entire DAVID enrichment network, doted box indicates region of magnification. Up regulated genes (colored nodes) connect to corresponding enriched functional annotations (grey nodes). (TIF) [file pgen.1007054.s002.tif]

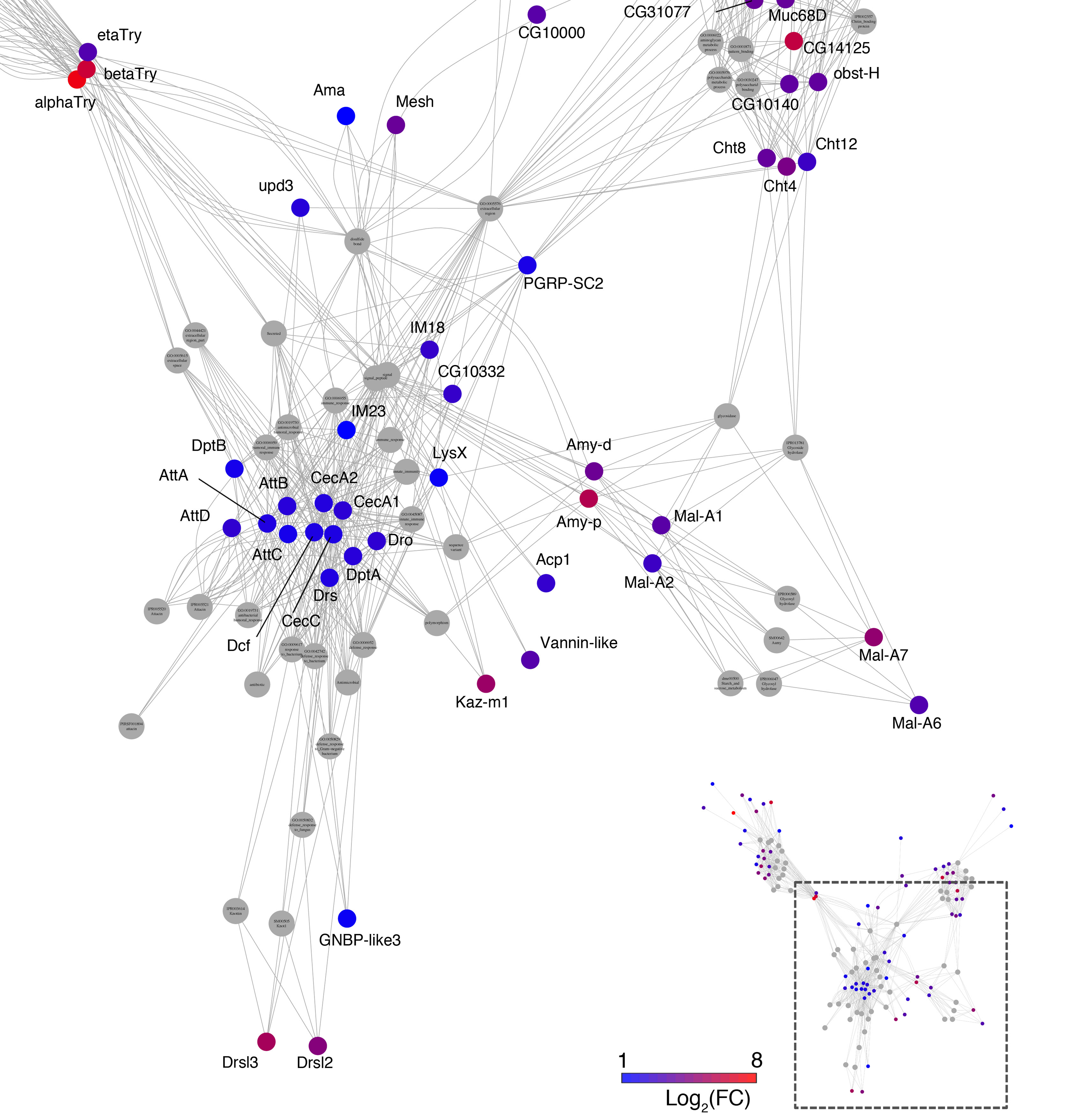

Supplement: S3 Fig — Inset illustrates the entire DAVID enrichment network, doted box indicates region of magnification. Up regulated genes (colored nodes) connect to corresponding enriched functional annotations (grey nodes). (TIF) [file pgen.1007054.s003.tif]
